# (*E*)-*N*-[4-(Di­ethyl­amino)-2-hy­droxy­benzyl­idene]-2,4,6-tri­methyl­benzenaminium nitrate

**DOI:** 10.1107/S2414314625002779

**Published:** 2025-04-04

**Authors:** Segun D. Oladipo, Sizwe J. Zamisa, Robert C. Luckay

**Affiliations:** a Department of Chemistry and Polymer Science, University of Stellenbosch, Private, Bag X1, Matieland, Stellenbosch 7602, South Africa; b School of Chemistry and Physics, University of KwaZulu-Natal, Private Bag, X54001, Durban 4000, South Africa

**Keywords:** crystal structure, protonated Schiff base, salt, hydrogen-bonding

## Abstract

The crystal structure of the title salt, C_20_H_27_N_2_O^+^·NO_3_^−^, has a cationic (*E*)-mesityl-*N*-[4-(di­ethyl­amino)­benzyl­idene]benzenaminium species and a nitrate counter-ion in the asymmetric-unit. In the crystal, alternating inter­molecular O—H⋯O and C—H⋯O hydrogen-bonding occurs between neighbouring protonated Schiff bases and nitrate ions within a supra­molecular, chain-like architecture that extends along the crystallographic *b*-axis direction.

## Abstract 2

The crystal structure of a protonated Schiff base, isolated as its nitrate salt, exhibits a supra­molecular chain-like architecture that propagates along the crystallographic *b*-axis direction and features inter­molecular O—H⋯O and C—H⋯O hydrogen-bonding.

## Graphical abstract

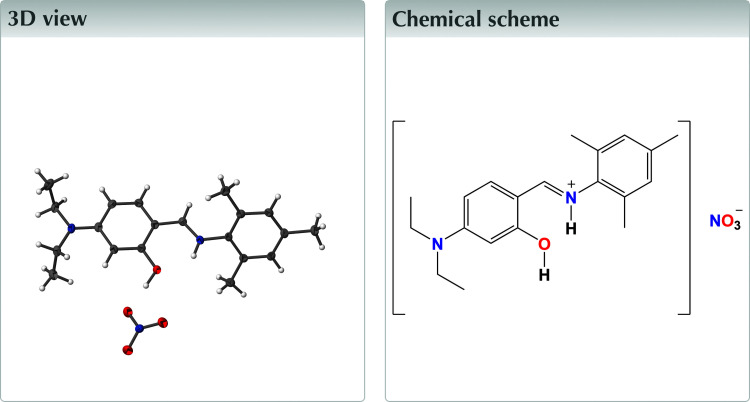

## Structure description

Schiff bases are well-known organic mol­ecules characterized by their ease of preparation, great pharmaceutical potential and for a myriad of applications (Adeleke *et al.*, 2024 ▸). In medicine, Schiff bases have been tested as anti-oxidants (Oladipo *et al.*, 2021 ▸) and anti-fungal agents (Jarrahpour *et al.*, 2004 ▸), among others (Thakur *et al.*, 2024 ▸) and their medicinal activities have been linked to the ability of the imine functional group to strongly bind to the nucleophilic or electrophilic moieties located in the active sites of enzymes. In this work, the crystal structure of the title protonated Schiff base, isolated as its nitrate salt, is reported.

**Figure 1:**
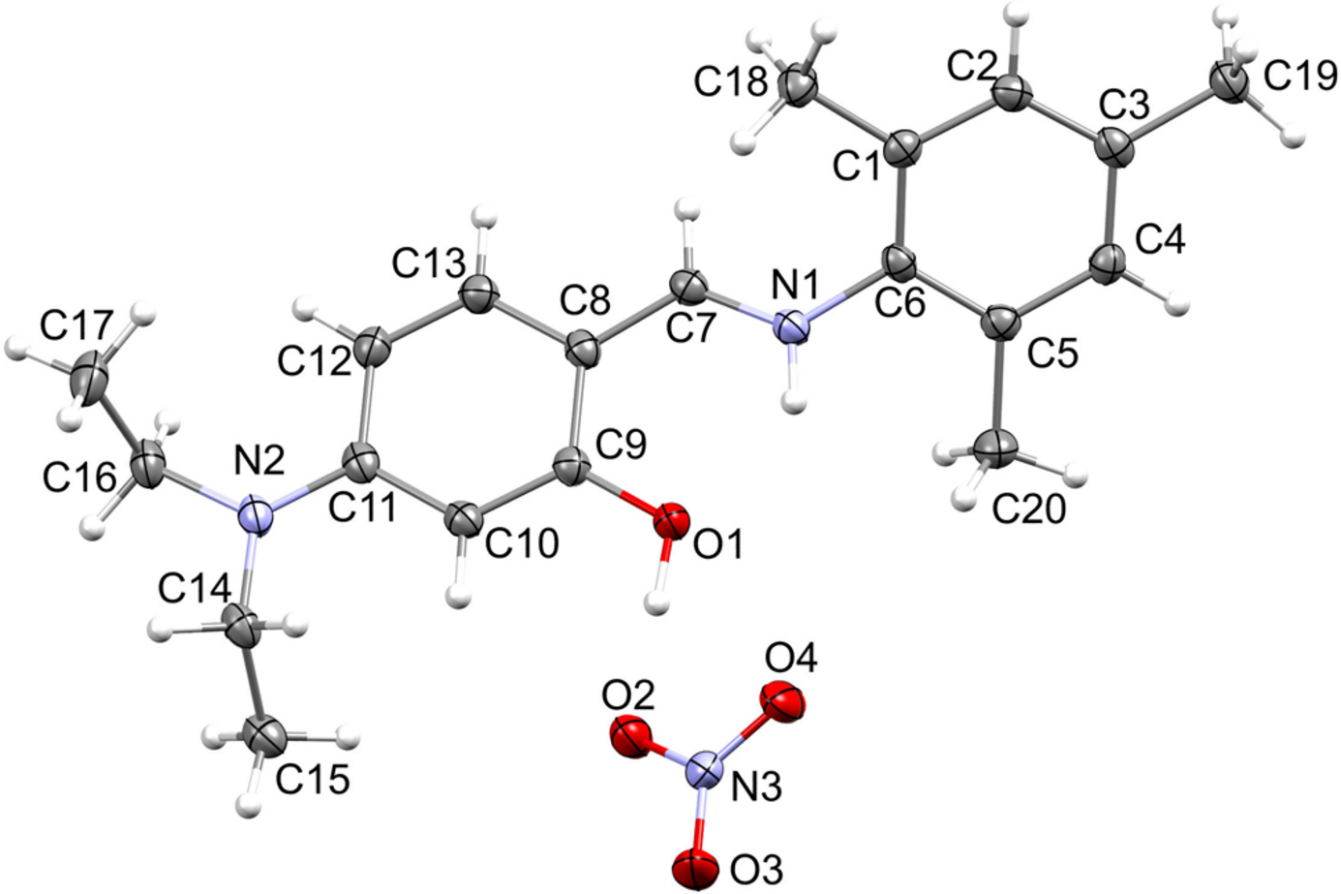
The mol­ecular structure of the asymmetric unit of the title salt showing the atom labelling and with ellipsoids drawn at the 50% probability level.

**Figure 2:**
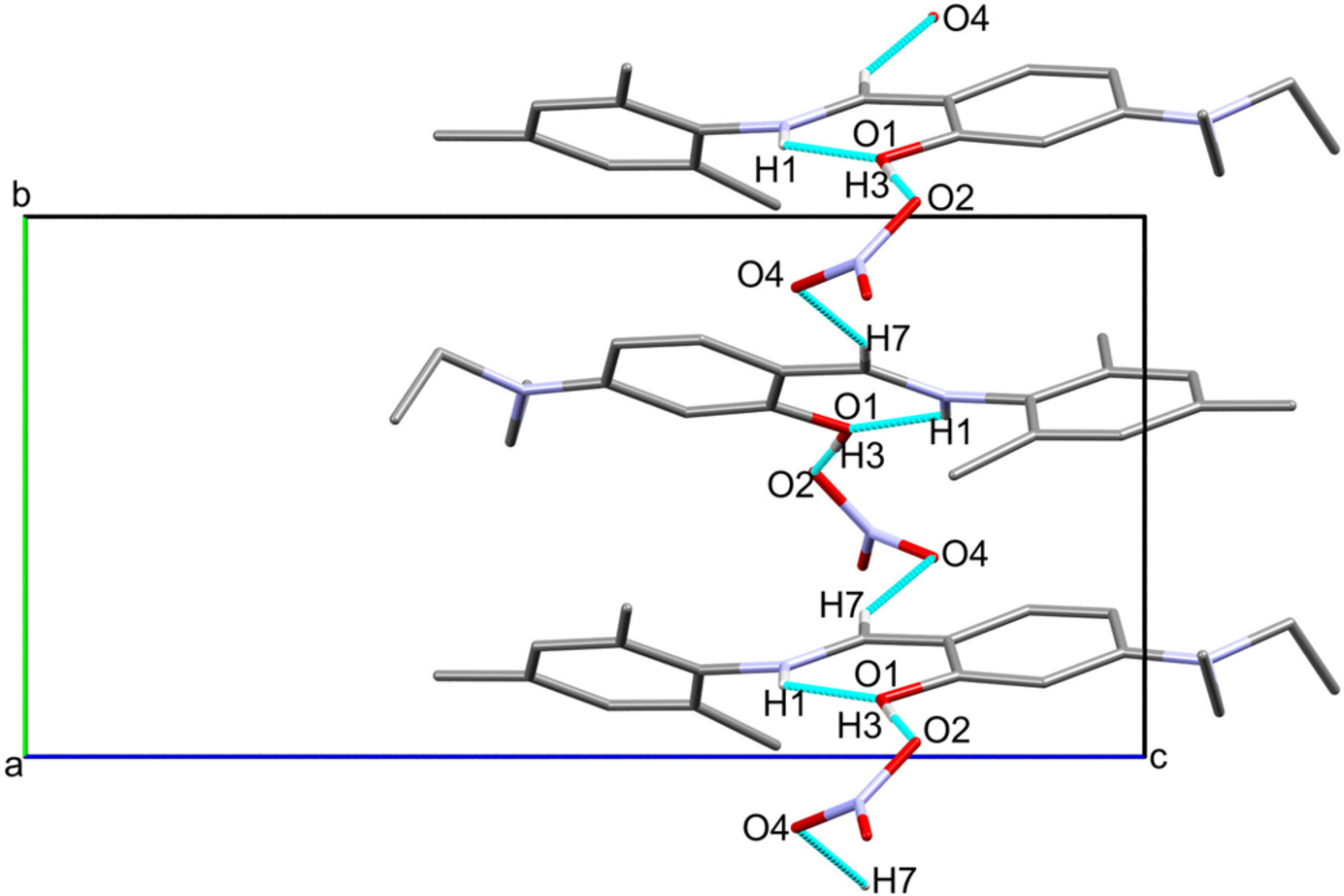
Representation of intra­molecular N1—H1⋯O1 hydrogen bonds and inter­molecular O1—H3⋯O2 and C7—H7⋯O4 hydrogen-bonding in the packing of the title salt. The hydrogen bonds are shown as turquoise bonds.

The structural analysis of the title salt revealed that its asymmetric unit contains an (*E*)-mesityl-*N*-[4-(di­ethyl­amino)­benzyl­idene]benzenaminium cation and a nitrate counter-anion (Fig. 1 ▸). The dihedral angle between the phenyl rings in the protonated Schiff base is 45.60 (1)°. This is substanti­ally wider than in the recently reported (*E*)-4-bromo-*N*-[4-(di­ethyl­amino)-2-hy­droxy­benzyl­idene]benzenaminium acetate-4-bromo­aniline [11.7 (1)°; Oladipo *et al.*, 2024 ▸] and similar to the one reported for the neutral Schiff base (*E*)-5-(di­ethyl­amino)-2-(phenyl­imino)­meth­yl)phenol [42.90 (1)°; Ranjith *et al.*, 2014 ▸]. There is an intra­molecular N—H⋯O hydrogen bond between the iminium H1 atom and the adjacent OH group (Table 1 ▸), as seen in similar compounds (protonated and neutral Schiff bases) (Oladipo *et al.*, 2024 ▸; Albayrak *et al.*, 2012 ▸). In the crystal, inter­molecular O—H⋯O hydrogen-bonding is observed between the H3 atom of the hydroxyl group of the protonated Schiff base and the O2 atom of the nitrate anion (Table 1 ▸). The anion further inter­acts with another protonated Schiff base mol­ecule *via* a C7—H7⋯O4 hydrogen bond (Table 1 ▸). Linking neighbouring mol­ecules in this manner occurs within a supra­molecular chain-like pattern that extends along the crystallographic *b*-axis direction as shown in Fig. 2 ▸.

## Synthesis and crystallization

The title compound was obtained during an attempt to prepare a binuclear, square-pyramidal binuclear copper(II) complex [Cu**L**NO_3_]_2_ where **L** = (*E*)-5-(di­ethyl­amino)-2-[(mesityl­imino)­meth­yl]phenol. The copper(II) complex was prepared by reacting copper(II) nitrate trihydrate (0.070 g, 0.300 mmol) with compound **L**H (0.100 g, 0.300 mmol) in methanol in a 1:1 ratio. The resulting mixture was stirred at room temperature for 5 h to afford a dark-brown precipitate, which was washed thoroughly with diethyl ether. The di­chloro­methane solution of the resulting complex was refluxed at 100°C. The hot solution of the complex was slowly evaporated for three days, and this afforded a dark-brown precipitate with a few yellow needles of the title salt as revealed by single-crystal X-ray diffraction analysis. The spectroscopic data for the neutral mol­ecule of **L**H have been previously reported (Oladipo & Luckay, 2025 ▸).

## Refinement

For full experimental details including crystal data, data collection and structure refinement details, refer to Table 2 ▸.

## Supplementary Material

Crystal structure: contains datablock(s) . DOI: 10.1107/S2414314625002779/tk4117sup1.cif

Structure factors: contains datablock(s) I. DOI: 10.1107/S2414314625002779/tk4117Isup2.hkl

Supporting information file. DOI: 10.1107/S2414314625002779/tk4117Isup3.cml

CCDC reference: 2412828

Additional supporting information:  crystallographic information; 3D view; checkCIF report

## Figures and Tables

**Table 1:** Hydrogen-bond geometry (Å, °)

*D*—H⋯*A*	*D*—H	H⋯*A*	*D*⋯*A*	*D*—H⋯*A*
N1—H1⋯O1	0.887 (19)	1.961 (19)	2.6410 (17)	132.3 (16)
O1—H3⋯O2	0.98 (2)	1.59 (2)	2.5673 (16)	178 (2)
C7—H7⋯O4^i^	0.95	2.47	3.359 (2)	157

Symmetry code: (i) 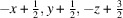.

**Table 2:** Experimental details

Crystal data	
Chemical formula	C_20_H_27_N_2_O^+^·NO_3_^−^
*M*_r_	373.44
Crystal system, space group	Monoclinic, *P*2_1_/*n*
Temperature (K)	100
*a*, *b*, *c* (Å)	15.3627 (16), 7.8529 (8), 16.9991 (17)
β (°)	106.424 (2)
*V* (Å^3^)	1967.1 (3)
*Z*	4
Radiation type	Mo *K*α
μ (mm^−1^)	0.09
Crystal size (mm)	0.36 × 0.12 × 0.06

Data collection	
Diffractometer	Bruker SMART APEX CCD
Absorption correction	Multi-scan (*SADABS*; Krause *et al.*, 2015 ▸)
*T*_min_, *T*_max_	0.912, 1.000
No. of measured, independent and observed [*I* > 2σ(*I*)] reflections	39793, 4524, 3387
*R*_int_	0.056
(sin θ/λ)_max_ (Å^−1^)	0.650

Refinement	
*R*[*F*^2^ > 2σ(*F*^2^)], *wR*(*F*^2^), *S*	0.044, 0.123, 1.04
No. of reflections	4524
No. of parameters	257
H-atom treatment	H atoms treated by a mixture of independent and constrained refinement
Δρ_max_, Δρ_min_ (e Å^−3^)	0.27, −0.24

Computer programs: *APEX4* (Bruker, 2021 ▸), *SAINT* (Bruker, 2019 ▸), *SHELXT2018/2* (Sheldrick, 2015*a* ▸), *SHELXL2018/3* (Sheldrick, 2015*b* ▸) and *X-SEED* (Barbour, 2020 ▸).

## full crystallographic data

### (E)-N-[4-(Diethylamino)-2-hydroxybenzylidene]-2,4,6-trimethylbenzenaminium nitrate Crystal data

**Table:**

C_20_H_27_N_2_O^+^·NO_3_^−^	*F*(000) = 800
*M**_r_* = 373.44	*D*_x_ = 1.261 Mg m^−^^3^
Monoclinic, *P*2_1_/*n*	Mo *K*α radiation, λ = 0.71073 Å
*a* = 15.3627 (16) Å	Cell parameters from 5347 reflections
*b* = 7.8529 (8) Å	θ = 2.8–26.9°
*c* = 16.9991 (17) Å	µ = 0.09 mm^−^^1^
β = 106.424 (2)°	*T* = 100 K
*V* = 1967.1 (3) Å^3^	Needle, yellow
*Z* = 4	0.36 × 0.12 × 0.06 mm

### (E)-N-[4-(Diethylamino)-2-hydroxybenzylidene]-2,4,6-trimethylbenzenaminium nitrate Data collection

**Table:**

Bruker SMART APEX CCD diffractometer	3387 reflections with *I* > 2σ(*I*)
Radiation source: microfocus sealed tube	*R*_int_ = 0.056
φ and ω scans	θ_max_ = 27.5°, θ_min_ = 2.1°
Absorption correction: multi-scan (SADABS; Krause *et al.*, 2015)	*h* = −19→19
*T*_min_ = 0.912, *T*_max_ = 1.000	*k* = −10→10
39793 measured reflections	*l* = −22→22
4524 independent reflections	

### (E)-N-[4-(Diethylamino)-2-hydroxybenzylidene]-2,4,6-trimethylbenzenaminium nitrate Refinement

**Table:**

Refinement on *F*^2^	Primary atom site location: intrinsic phasing
Least-squares matrix: full	Secondary atom site location: difference Fourier map
*R*[*F*^2^ > 2σ(*F*^2^)] = 0.044	Hydrogen site location: mixed
*wR*(*F*^2^) = 0.123	H atoms treated by a mixture of independent and constrained refinement
*S* = 1.04	*w* = 1/[σ^2^(*F*_o_^2^) + (0.0526*P*)^2^ + 0.9367*P*] where *P* = (*F*_o_^2^ + 2*F*_c_^2^)/3
4524 reflections	(Δ/σ)_max_ < 0.001
257 parameters	Δρ_max_ = 0.27 e Å^−^^3^
0 restraints	Δρ_min_ = −0.24 e Å^−^^3^

### (E)-N-[4-(Diethylamino)-2-hydroxybenzylidene]-2,4,6-trimethylbenzenaminium nitrate Special details

**Table:**

Geometry. All esds (except the esd in the dihedral angle between two l.s. planes) are estimated using the full covariance matrix. The cell esds are taken into account individually in the estimation of esds in distances, angles and torsion angles; correlations between esds in cell parameters are only used when they are defined by crystal symmetry. An approximate (isotropic) treatment of cell esds is used for estimating esds involving l.s. planes.

### (E)-N-[4-(Diethylamino)-2-hydroxybenzylidene]-2,4,6-trimethylbenzenaminium nitrate Fractional atomic coordinates and isotropic or equivalent isotropic displacement parameters (Å^2^)

**Table:**

	*x*	*y*	*z*	*U*_iso_*/*U*_eq_	
O1	0.36159 (7)	0.60622 (15)	0.73553 (6)	0.0233 (3)	
O2	0.51066 (8)	0.52660 (17)	0.70465 (7)	0.0311 (3)	
O4	0.52064 (8)	0.36875 (19)	0.81118 (8)	0.0378 (3)	
N1	0.24624 (9)	0.66708 (17)	0.82160 (8)	0.0201 (3)	
O3	0.62397 (8)	0.3541 (2)	0.74825 (8)	0.0448 (4)	
N2	0.26047 (9)	0.67856 (17)	0.44476 (8)	0.0221 (3)	
N3	0.55298 (9)	0.41504 (19)	0.75557 (8)	0.0256 (3)	
C6	0.22393 (10)	0.66044 (19)	0.89762 (9)	0.0183 (3)	
C9	0.29748 (10)	0.6504 (2)	0.66634 (9)	0.0194 (3)	
C5	0.29141 (10)	0.7121 (2)	0.96781 (9)	0.0198 (3)	
C8	0.21396 (10)	0.7139 (2)	0.67550 (9)	0.0195 (3)	
C7	0.19268 (10)	0.71869 (19)	0.75056 (9)	0.0197 (3)	
H7	0.134953	0.762772	0.750103	0.024*	
C10	0.31165 (10)	0.6372 (2)	0.59033 (9)	0.0203 (3)	
H10	0.366796	0.589523	0.585615	0.024*	
C1	0.14072 (10)	0.59584 (19)	0.90178 (9)	0.0195 (3)	
C11	0.24554 (10)	0.69324 (19)	0.51894 (9)	0.0202 (3)	
C4	0.27264 (10)	0.7048 (2)	1.04296 (9)	0.0203 (3)	
H4	0.318012	0.738307	1.091112	0.024*	
C3	0.18878 (10)	0.6494 (2)	1.04934 (9)	0.0208 (3)	
C12	0.16344 (10)	0.7649 (2)	0.52836 (9)	0.0226 (3)	
H12	0.118870	0.807924	0.481811	0.027*	
C13	0.14887 (10)	0.7719 (2)	0.60350 (9)	0.0217 (3)	
H13	0.093131	0.817191	0.607995	0.026*	
C2	0.12505 (10)	0.5950 (2)	0.97867 (9)	0.0208 (3)	
H2	0.068073	0.555398	0.982781	0.025*	
C18	0.06990 (11)	0.5214 (2)	0.82955 (10)	0.0247 (4)	
H18A	0.036626	0.431377	0.848651	0.037*	
H18B	0.099560	0.473479	0.790515	0.037*	
H18C	0.027526	0.611001	0.802565	0.037*	
C14	0.33831 (11)	0.5834 (2)	0.43356 (10)	0.0242 (3)	
H14A	0.322260	0.538199	0.376900	0.029*	
H14B	0.350223	0.484913	0.471474	0.029*	
C19	0.16725 (11)	0.6500 (2)	1.13067 (10)	0.0265 (4)	
H19A	0.118183	0.731138	1.128528	0.040*	
H19B	0.221392	0.683433	1.174201	0.040*	
H19C	0.148204	0.535704	1.142072	0.040*	
C16	0.19707 (11)	0.7476 (2)	0.37022 (9)	0.0260 (4)	
H16A	0.230934	0.775748	0.330283	0.031*	
H16B	0.170520	0.854459	0.384064	0.031*	
C20	0.38255 (11)	0.7745 (2)	0.96248 (10)	0.0274 (4)	
H20A	0.420502	0.804711	1.017416	0.041*	
H20B	0.373998	0.874901	0.926880	0.041*	
H20C	0.412310	0.684327	0.939695	0.041*	
C15	0.42461 (11)	0.6876 (2)	0.44842 (10)	0.0267 (4)	
H15A	0.412978	0.788083	0.412813	0.040*	
H15B	0.472149	0.618124	0.436134	0.040*	
H15C	0.444467	0.723744	0.505913	0.040*	
C17	0.12084 (12)	0.6248 (2)	0.33054 (10)	0.0333 (4)	
H17A	0.146615	0.518840	0.316435	0.050*	
H17B	0.081295	0.676321	0.280665	0.050*	
H17C	0.085442	0.600040	0.368949	0.050*	
H1	0.3015 (13)	0.633 (2)	0.8224 (11)	0.031 (5)*	
H3	0.4180 (16)	0.573 (3)	0.7241 (14)	0.059 (7)*	

### (E)-N-[4-(Diethylamino)-2-hydroxybenzylidene]-2,4,6-trimethylbenzenaminium nitrate Atomic displacement parameters (Å^2^)

**Table:**

	*U*^11^	*U*^22^	*U*^33^	*U*^12^	*U*^13^	*U*^23^
O1	0.0204 (5)	0.0323 (7)	0.0179 (5)	0.0038 (5)	0.0065 (4)	0.0024 (5)
O2	0.0293 (6)	0.0409 (8)	0.0255 (6)	0.0075 (5)	0.0117 (5)	0.0065 (5)
O4	0.0266 (6)	0.0605 (9)	0.0292 (7)	0.0037 (6)	0.0127 (5)	0.0153 (6)
N1	0.0203 (7)	0.0229 (7)	0.0186 (6)	0.0023 (5)	0.0081 (5)	0.0000 (5)
O3	0.0248 (6)	0.0820 (11)	0.0288 (7)	0.0200 (7)	0.0096 (5)	0.0059 (7)
N2	0.0256 (7)	0.0251 (7)	0.0160 (6)	−0.0032 (6)	0.0067 (5)	−0.0011 (5)
N3	0.0183 (6)	0.0390 (9)	0.0186 (6)	−0.0009 (6)	0.0035 (5)	−0.0023 (6)
C6	0.0214 (7)	0.0175 (7)	0.0173 (7)	0.0035 (6)	0.0076 (6)	0.0009 (6)
C9	0.0206 (7)	0.0185 (8)	0.0190 (7)	−0.0006 (6)	0.0055 (6)	0.0005 (6)
C5	0.0199 (7)	0.0190 (8)	0.0209 (7)	0.0024 (6)	0.0066 (6)	0.0007 (6)
C8	0.0218 (7)	0.0191 (8)	0.0186 (7)	−0.0018 (6)	0.0074 (6)	0.0003 (6)
C7	0.0204 (7)	0.0189 (8)	0.0206 (7)	0.0003 (6)	0.0071 (6)	−0.0008 (6)
C10	0.0215 (7)	0.0206 (8)	0.0203 (7)	−0.0015 (6)	0.0083 (6)	−0.0019 (6)
C1	0.0201 (7)	0.0172 (8)	0.0207 (7)	0.0030 (6)	0.0051 (6)	0.0021 (6)
C11	0.0249 (8)	0.0175 (7)	0.0188 (7)	−0.0052 (6)	0.0074 (6)	−0.0023 (6)
C4	0.0225 (7)	0.0194 (8)	0.0179 (7)	0.0024 (6)	0.0036 (6)	−0.0010 (6)
C3	0.0257 (8)	0.0182 (8)	0.0204 (7)	0.0050 (6)	0.0095 (6)	0.0022 (6)
C12	0.0228 (8)	0.0242 (8)	0.0186 (7)	−0.0010 (6)	0.0026 (6)	0.0009 (6)
C13	0.0200 (7)	0.0230 (8)	0.0224 (8)	0.0002 (6)	0.0064 (6)	−0.0011 (6)
C2	0.0193 (7)	0.0209 (8)	0.0235 (8)	0.0019 (6)	0.0080 (6)	0.0032 (6)
C18	0.0253 (8)	0.0257 (9)	0.0222 (8)	−0.0031 (7)	0.0051 (6)	0.0018 (6)
C14	0.0313 (8)	0.0235 (8)	0.0203 (8)	−0.0025 (7)	0.0114 (7)	−0.0037 (6)
C19	0.0298 (8)	0.0304 (9)	0.0217 (8)	0.0019 (7)	0.0115 (7)	0.0022 (7)
C16	0.0335 (9)	0.0281 (9)	0.0163 (7)	−0.0031 (7)	0.0071 (7)	0.0010 (6)
C20	0.0215 (8)	0.0351 (10)	0.0262 (8)	−0.0032 (7)	0.0076 (7)	−0.0016 (7)
C15	0.0300 (9)	0.0268 (9)	0.0270 (8)	−0.0028 (7)	0.0139 (7)	−0.0023 (7)
C17	0.0380 (10)	0.0367 (10)	0.0198 (8)	−0.0067 (8)	−0.0003 (7)	0.0003 (7)

### (E)-N-[4-(Diethylamino)-2-hydroxybenzylidene]-2,4,6-trimethylbenzenaminium nitrate Geometric parameters (Å, º)

**Table:**

O1—C9	1.3485 (18)	C3—C2	1.385 (2)
O1—H3	0.98 (2)	C3—C19	1.510 (2)
O2—N3	1.2733 (18)	C12—C13	1.359 (2)
O4—N3	1.2402 (17)	C12—H12	0.9500
N1—C7	1.318 (2)	C13—H13	0.9500
N1—C6	1.4285 (19)	C2—H2	0.9500
N1—H1	0.887 (19)	C18—H18A	0.9800
O3—N3	1.2293 (18)	C18—H18B	0.9800
N2—C11	1.3489 (19)	C18—H18C	0.9800
N2—C16	1.466 (2)	C14—C15	1.517 (2)
N2—C14	1.468 (2)	C14—H14A	0.9900
C6—C1	1.396 (2)	C14—H14B	0.9900
C6—C5	1.401 (2)	C19—H19A	0.9800
C9—C10	1.374 (2)	C19—H19B	0.9800
C9—C8	1.426 (2)	C19—H19C	0.9800
C5—C4	1.388 (2)	C16—C17	1.520 (2)
C5—C20	1.510 (2)	C16—H16A	0.9900
C8—C7	1.404 (2)	C16—H16B	0.9900
C8—C13	1.419 (2)	C20—H20A	0.9800
C7—H7	0.9500	C20—H20B	0.9800
C10—C11	1.415 (2)	C20—H20C	0.9800
C10—H10	0.9500	C15—H15A	0.9800
C1—C2	1.394 (2)	C15—H15B	0.9800
C1—C18	1.509 (2)	C15—H15C	0.9800
C11—C12	1.431 (2)	C17—H17A	0.9800
C4—C3	1.393 (2)	C17—H17B	0.9800
C4—H4	0.9500	C17—H17C	0.9800
			
C9—O1—H3	111.5 (14)	C3—C2—C1	122.99 (14)
C7—N1—C6	126.34 (13)	C3—C2—H2	118.5
C7—N1—H1	117.0 (12)	C1—C2—H2	118.5
C6—N1—H1	116.7 (12)	C1—C18—H18A	109.5
C11—N2—C16	121.95 (13)	C1—C18—H18B	109.5
C11—N2—C14	121.66 (13)	H18A—C18—H18B	109.5
C16—N2—C14	116.28 (12)	C1—C18—H18C	109.5
O3—N3—O4	121.41 (15)	H18A—C18—H18C	109.5
O3—N3—O2	119.75 (14)	H18B—C18—H18C	109.5
O4—N3—O2	118.84 (13)	N2—C14—C15	114.18 (13)
C1—C6—C5	121.99 (13)	N2—C14—H14A	108.7
C1—C6—N1	121.11 (13)	C15—C14—H14A	108.7
C5—C6—N1	116.80 (13)	N2—C14—H14B	108.7
O1—C9—C10	122.02 (14)	C15—C14—H14B	108.7
O1—C9—C8	116.91 (13)	H14A—C14—H14B	107.6
C10—C9—C8	121.07 (14)	C3—C19—H19A	109.5
C4—C5—C6	118.40 (14)	C3—C19—H19B	109.5
C4—C5—C20	120.39 (14)	H19A—C19—H19B	109.5
C6—C5—C20	121.21 (13)	C3—C19—H19C	109.5
C7—C8—C13	119.11 (14)	H19A—C19—H19C	109.5
C7—C8—C9	123.83 (14)	H19B—C19—H19C	109.5
C13—C8—C9	117.05 (13)	N2—C16—C17	112.90 (14)
N1—C7—C8	125.35 (14)	N2—C16—H16A	109.0
N1—C7—H7	117.3	C17—C16—H16A	109.0
C8—C7—H7	117.3	N2—C16—H16B	109.0
C9—C10—C11	121.15 (14)	C17—C16—H16B	109.0
C9—C10—H10	119.4	H16A—C16—H16B	107.8
C11—C10—H10	119.4	C5—C20—H20A	109.5
C2—C1—C6	116.91 (14)	C5—C20—H20B	109.5
C2—C1—C18	119.02 (14)	H20A—C20—H20B	109.5
C6—C1—C18	124.02 (14)	C5—C20—H20C	109.5
N2—C11—C10	120.32 (14)	H20A—C20—H20C	109.5
N2—C11—C12	121.76 (14)	H20B—C20—H20C	109.5
C10—C11—C12	117.92 (13)	C14—C15—H15A	109.5
C5—C4—C3	121.46 (14)	C14—C15—H15B	109.5
C5—C4—H4	119.3	H15A—C15—H15B	109.5
C3—C4—H4	119.3	C14—C15—H15C	109.5
C2—C3—C4	118.12 (14)	H15A—C15—H15C	109.5
C2—C3—C19	120.72 (14)	H15B—C15—H15C	109.5
C4—C3—C19	121.16 (14)	C16—C17—H17A	109.5
C13—C12—C11	120.38 (14)	C16—C17—H17B	109.5
C13—C12—H12	119.8	H17A—C17—H17B	109.5
C11—C12—H12	119.8	C16—C17—H17C	109.5
C12—C13—C8	122.32 (14)	H17A—C17—H17C	109.5
C12—C13—H13	118.8	H17B—C17—H17C	109.5
C8—C13—H13	118.8		
			
C7—N1—C6—C1	44.6 (2)	C16—N2—C11—C12	5.1 (2)
C7—N1—C6—C5	−139.14 (16)	C14—N2—C11—C12	−171.08 (14)
C1—C6—C5—C4	−2.7 (2)	C9—C10—C11—N2	−179.71 (14)
N1—C6—C5—C4	−178.99 (14)	C9—C10—C11—C12	0.2 (2)
C1—C6—C5—C20	177.30 (15)	C6—C5—C4—C3	−0.6 (2)
N1—C6—C5—C20	1.0 (2)	C20—C5—C4—C3	179.33 (15)
O1—C9—C8—C7	−5.6 (2)	C5—C4—C3—C2	2.4 (2)
C10—C9—C8—C7	175.08 (15)	C5—C4—C3—C19	−176.95 (15)
O1—C9—C8—C13	175.59 (14)	N2—C11—C12—C13	177.41 (15)
C10—C9—C8—C13	−3.7 (2)	C10—C11—C12—C13	−2.5 (2)
C6—N1—C7—C8	−175.65 (15)	C11—C12—C13—C8	1.7 (2)
C13—C8—C7—N1	179.16 (15)	C7—C8—C13—C12	−177.45 (15)
C9—C8—C7—N1	0.4 (3)	C9—C8—C13—C12	1.4 (2)
O1—C9—C10—C11	−176.33 (14)	C4—C3—C2—C1	−1.0 (2)
C8—C9—C10—C11	2.9 (2)	C19—C3—C2—C1	178.41 (15)
C5—C6—C1—C2	4.1 (2)	C6—C1—C2—C3	−2.2 (2)
N1—C6—C1—C2	−179.83 (13)	C18—C1—C2—C3	175.28 (15)
C5—C6—C1—C18	−173.26 (15)	C11—N2—C14—C15	−86.67 (18)
N1—C6—C1—C18	2.8 (2)	C16—N2—C14—C15	96.94 (16)
C16—N2—C11—C10	−174.94 (14)	C11—N2—C16—C17	−86.32 (19)
C14—N2—C11—C10	8.9 (2)	C14—N2—C16—C17	90.06 (17)

### (E)-N-[4-(Diethylamino)-2-hydroxybenzylidene]-2,4,6-trimethylbenzenaminium nitrate Hydrogen-bond geometry (Å, º)

**Table:**

*D*—H···*A*	*D*—H	H···*A*	*D*···*A*	*D*—H···*A*
N1—H1···O1	0.887 (19)	1.961 (19)	2.6410 (17)	132.3 (16)
O1—H3···O2	0.98 (2)	1.59 (2)	2.5673 (16)	178 (2)
C7—H7···O4^i^	0.95	2.47	3.359 (2)	157

Symmetry code: (i) −*x*+1/2, *y*+1/2, −*z*+3/2.
